# Drug-induced mitochondrial impairment in liver cells

**DOI:** 10.17179/excli2015-764

**Published:** 2015-12-22

**Authors:** Regina Stöber

**Affiliations:** 1 Leibniz Research Centre for Working Environment and Human Factors at TU Dortmund (IfADo), Ardeystrasse 67, 44139 Dortmund, Germany

## ⁯

Recently, Laia Tolosa and colleagues from Valencia University have published a study how to identify compounds that cause hepatotoxicity due to compromising mitochondrial functions (Tolosa et al., 2015[23]). The authors used a set of fluorescent probes for imaging: HepG2 cells were loaded with Hoechst 33342 for cell number determination, cell viability was determined with propidium iodid and Mitotracker Green FM was applied for quantification of mitochondrial mass (Tolosa et al., 2015[23]). For a more detailed analysis of compromised mitochondria, the authors used Fluo-4 AM to detect changes in cytoplasmic free calcium, Mitotracker Deep Red for analysis of mitochondrial localization, MitoSOX Red to analyze generation of superoxide by mitochondria, TMRM to study mitochondrial membrane potential and YO-PRO-1 for detection of apoptotic cells. Using this set of markers and quantitative imaging techniques the authors classified a set of well-known mitochondrial hepatotoxicants with excellent accuracy (Tolosa et al., 2015[23]).

Drug or chemically induced liver injury represents a cutting-edge topic in toxicology (Benet et al., 2014[1]; Campos et al., 2014[2]; Vitins et al., 2014[25]; Liu et al., 2014[16]; Godoy and Bolt, 2012[6]; Vinken et al., 2012[24]; Liang et al., 2011[15]; Hammad et al., 2011[11]). Numerous research projects aim at establishing *in vitro* systems to predict human hepatotoxicity (Chen et al., 2014[4]; Grinberg et al., 2014[10]; Carvalho et al., 2004[3]; O'Brien et al., 2006[17]; Reif, 2015[19]; Shinde et al., 2015[21][22]; Kim et al., 2015[14]; Pfeiffer et al., 2015[18]). One of the limitations of current research is that it still remains challenging to predict doses that are associated with an increased risk of hepatotoxicity *in vivo* (Ghallab, 2013[5]; Reif, 2014[20]). Moreover, cultivated cells undergo major changes compared to hepatocytes in an intact liver (Godoy et al., 2015[9], 2013[8], 2009[7]; Zellmer et al., 2010[26]; Hewitt et al., 2007[13]; Hengstler et al., 2000[12]). Despite of the still remaining challenges the high-content screening platform established by Tolosa and colleagues represents an important milestone.
